# XIIIth International Medical Congress

**Published:** 1899-12

**Authors:** Henry Barton Jacobs

**Affiliations:** Secretary American National Committee; 3 West Franklin St., Baltimore, Md.


### XIIITH INTERNATIONAL MEDICAL CONGRESS.

### AMERICAN NATIONAL COMMITTEE.

BALTIMORE, MD., November 1st, 1899.

DEAR DOCTOR:—The American National Committee of the XIIIth International Medical Congress, to be held in Paris, from the 2d to the 9th of August, 1900, in connection with the French Exposition, has been organized as above indicated.

All doctors of medicine are entitled to membership in this congress by making the proper application and paying the sum of $5. The secretary-general in Paris has instructed the American National Committee to receive the applications of American physicians, and for this purpose a blank form is enclosed, upon which is to be written full name and address, degrees and any position of note held, together with the section of the congress to which the writer wishes to belong. A visiting card should also be appended. These forms, with the $5, are to be returned to the secretary of the national committee. He in turn will send receipt and forward slips and money to Paris, where they will be registered, and in due course of time a card of admission to the congress mailed to each applicant.

The committee hopes the American representation in this extremely important medical congress may be as large as possible, and they would urge every member of the profession to enter his name for membership, this alone entitling him to receive a digest of the full proceedings of the congress and the printed report of the section to which he belongs.

Very sincerely yours,
Henry Barton Jacobs,
*Secretary American National Committee.*

3 West Franklin St., Baltimore, Md.

(Physicians should write to Dr. Jacobs for a copy of the circular and letter from which full information can be obtained.—Ed.)

